# RSV Induces Activation of Intracellular EGFR on the Mitochondrial Membrane for Virus Propagation

**DOI:** 10.3390/ijms242417431

**Published:** 2023-12-13

**Authors:** Se Sil Noh, Hye Jin Shin

**Affiliations:** 1 Department of Microbiology, School of Medicine, Chungnam National University, Daejeon 35015, Republic of Korea; wotuz0123@o.cnu.ac.kr; 2 Department of Medical Science, School of Medicine, Chungnam National University, Daejeon 35015, Republic of Korea; 3 Brain Korea 21 FOUR Project for Medical Science, Chungnam National University, Daejeon 35015, Republic of Korea; 4 Research Institute for Medical Sciences, School of Medicine, Chungnam National University, Daejeon 35015, Republic of Korea

**Keywords:** intracellular epidermal growth factor receptor, vandetanib, respiratory syncytial virus, mitochondrial homeostasis

## Abstract

Respiratory syncytial virus (RSV) infects people of all ages and is one of the most common causative agents of lower respiratory tract infections, such as pneumonia, especially in infants under one year of age. However, no direct treatment has been developed for RSV infections. Maintenance of mitochondrial homeostasis and epidermal growth factor receptor (EGFR) activity is important for human cell growth. This study reported that RSV infection maintained the total cellular ATP levels and promoted the intracellular activity of EGFR to replicate RSV. RSV activates the intracellular EGFR-mediated cell survival signaling cascade and maintains mitochondrial EGFR expression for viral production during early events after infection. The approved EGFR inhibitor, vandetanib, markedly reduces RSV propagation, suggesting that EGFR is an attractive host target for RSV therapeutics. Our results suggest that RSV infection maintains cellular ATP levels and promotes the activation of intracellular EGFR in the mitochondrial membrane, significantly contributing to robust RSV propagation.

## 1. Introduction

The epidermal growth factor receptor (EGFR) protein, a 170 kDa transmembrane receptor, is a member of the ErbB family of receptor tyrosine kinases. It comprises three domains: an extracellular domain (ECD), a transmembrane domain, and an intracellular domain (ICD). The binding of the ECD to ligands such as EGF induces conformational changes for downstream signaling, as does stress. EGFR, which is expressed in the epithelial layers, is implicated in cancer and regulates cellular processes, including proliferation and apoptosis [1,2,3]. EGFR acts as a homeostatic regulator, orchestrating essential cellular processes, including proliferation, apoptosis inhibition, cell migration and differentiation, mucus production, activation of inflammatory response, and overall maintenance of cell survival [4]. Positioned prominently on the plasma membrane, EGFR exhibits ubiquitous expression across diverse intracellular organelles, including endosomes, mitochondria, the nucleus, and lysosomes; thereby underscoring its intricate involvement in cellular functions and signaling pathways [5]. Upon activation, EGFR undergoes dimerization and phosphorylation, followed by recruiting adaptors and activating downstream signaling pathways, such as MAPKs, JAK/STAT, and PI3Ks. Specific tyrosine residues have distinct roles in these processes [6,7]. EGFR internalization involves endosomal trafficking with recycling to the cell surface or translocation to intracellular organelles, such as the nucleus and mitochondria. Nuclear translocation activates DNA-dependent protein kinases and influences their transcriptional activity. In addition, translocation of EGFR into the mitochondria affects ATP and ROS generation, contributing to mitochondrial bioenergetics. The overall EGFR signaling network is crucial for cellular homeostasis, governing cell survival, and maintaining equilibrium [8,9,10].

EGFR is not only important for understanding diseases related to cell survival and proliferation but also plays a crucial role in understanding viral infections [1,11,12,13]. Viruses recognize and interact with EGFR as an indispensable component of their strategies for host cell entry [14]. The central role of EGFR is the regulation of viral entry, replication, and immune evasion. Notably, EGFR trafficking plays a pivotal role in virus–host interactions by translocating EGFR into an array of intracellular organelles, including endosomes, lysosomes, mitochondria, and even the nucleus [15]. The respiratory syncytial virus (RSV) significantly causes lower respiratory tract infections in children and exacerbates chronic lung diseases in adults. RSV, an enveloped virus with a single-stranded RNA genome, increases airway mucin expression during infection [16,17,18]. Despite its widespread occurrence, there are currently no efficacious antiviral drugs or approved vaccines against RSV. EGFR plays a crucial role in lung epithelial cells during RSV infection, influencing inflammatory responses and promoting cell survival [19,20]. However, the precise mechanisms underlying these phenomena remain elusive and require further investigation to delineate the intricate molecular pathways and regulatory elements governing EGFR-mediated modulation in the context of RSV infection. In this study, we observed the functions of EGFR in the mitochondria to maintain cell survival and viral replication during early RSV infection.

## 2. Results

### 2.1. RSV Maintains Intracellular ATP Levels and Cell Viability

**Figure 1:**
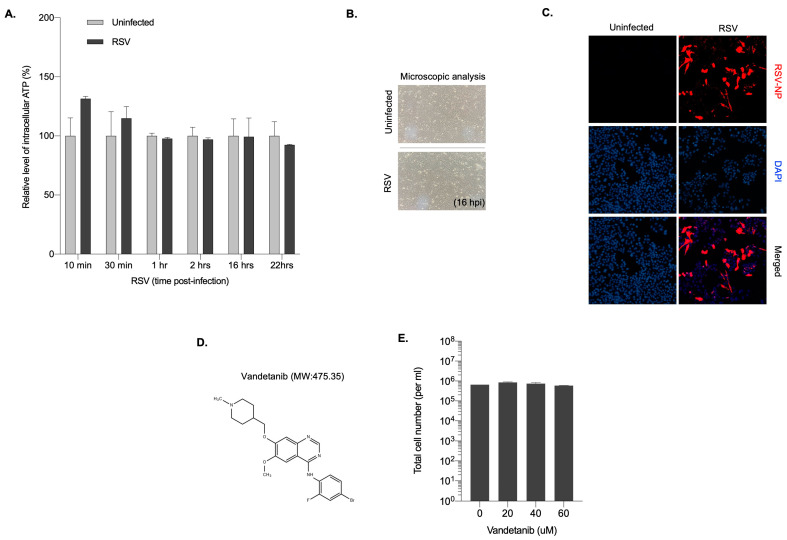
Cells maintain intracellular ATP levels early after RSV infection. (**A**) Quantification of intracellular ATP level in RSV infected HEp-2 cells at the indicated time points. Data are presented as the mean of duplicate experiments. (**B**) Light microscopy of RSV-infected HEp-2 cells at 16 hpi (200× magnification). (**C**) Immunofluorescence microscopy of RSV infectivity in HEp-2 cells. HEp-2 cells were infected with RSV at MOI 0.1 and immunostained with RSV nucleoprotein (NP) at 16 hpi. Nuclei, DAPI (blue); infection maker, RSV-NP antigen (red) (200× magnification). (**D**) The structure and molecular weight (MW) of vandetanib. (**E**) Viability quantification of vandetanib-treated HEp 2 cells. HEp-2 cells were treated with vandetanib at the indicated concentration for 16 h. Total cell number and cell viability were quantified using a hemocytometer and the Trypan Blue exclusion method.

To investigate intracellular ATP levels in the early stages of RSV infection, HEp-2 cell infected with RSV were used. Total intracellular ATP levels were quantified to reveal that, before 22 h post-infection (hpi), a period characterized by active viral replication, ATP levels exhibited a slight increase, subsequently plateauing and maintaining stability compared with uninfected cells. At 22 h post-infection, ATP levels were slightly decreased in RSV-infected cells (Figure 1A). The cellular response to RSV infection was further evaluated by microscopic examination of RSV-infected HEp-2 cells for 16 h to assess the cytopathic effects (CPE). Cell viability was maintained at 16 h post-infection, suggesting that the observed increase in ATP levels was not accompanied by a significant decrease in cell viability at that time point (Figure 1B). RSV infection at an MOI of 0.1 was also confirmed by immunostaining for RSV nucleoprotein (NP) at 16 hpi, with the red signal indicating RSV-infected cells (Figure 1C). These results suggested that 16 hpi was the optimal time point for elucidating the mechanisms underlying ATP maintenance during viral infection. Hypothesizing that EGFR activation plays a crucial role in preserving mitochondrial homeostasis and cellular growth to facilitate robust viral replication and sustain ATP expression during early RSV infection, we used vandetanib, an FDA-approved EGFR inhibitor (Figure 1D). The optimal concentration of vandetanib was determined by assessing cell viability in HEp-2 cells, revealing no severe cytotoxicity within the 20–60 μM concentration range (Figure 1E). These findings suggest a potential link among EGFR activation, mitochondrial homeostasis, and ATP maintenance during the early stages of RSV infection, prompting further exploration of the role of EGFR in viral replication.

### 2.2. Vandetanib Is a Potent Antiviral Agent against RSV Propagation

**Figure 2:**
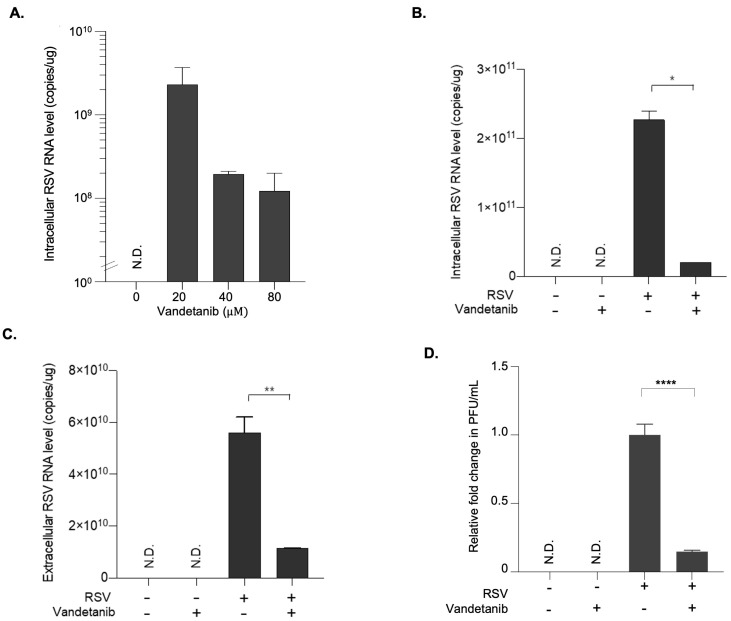
Antiviral effect of vandetanib against RSV infection. At 1 h post-infection, HEp-2 cells infected with RSV at an MOI of 0.1 were washed with fresh cell culture media and subsequently treated with the vandetanib for 15 h. Cell culture supernatant and pellet were used for further analyses. (**A**) Dose-dependent antiviral effect of vandetanib against RSV. Intracellular RNA isolated from RSV infected HEp 2 cells was used for the analysis of RSV RNA level by real time qRT PCR. (**B**) Intracellular RNA isolated from RSV-infected cells with vandetanib treatment (60 μM) was used for the analysis of RSV RNA levels using PCR primers specific to the RSV N gene. (**C**) Extracellular RNA isolated from culture supernatants of RSV-infected cells was used for the analysis of RSV RNA levels using PCR primers specific to the RSV N gene. (**D**) Relative fold change in the infectious viral titers of RSV in vandetanib treated samples compared against that treated with vehicle, represented as fold change in PFU (plaque forming units)/mL. The intracellular and extracellular RSV RNA copy numbers were determined by real time qRT-PCR. P-values were calculated by unpaired Student’s *t*-tests (mean ± SD; *n* = 2; * *p* ≤ 0.05, ** *p* ≤ 0.005, **** *p* ≤ 0.00005). N.D. (Not determined).

The dose-dependent antiviral efficacy of vandetanib against RSV infection was evaluated in HEp-2 cells at a concentration range of 20–60 μM. The qRT-PCR results revealed a dose-dependent reduction in intracellular RSV RNA copy numbers (Figure 2A). Specifically, a more than 10-fold decrease was observed with 60 μM vandetanib treatment compared to 20 μM. The inhibitory effect of vandetanib on RSV propagation was observed at both the intracellular and extracellular levels. HEp-2 cells, infected with RSV at a multiplicity of infection (MOI) of 0.1, were treated with vandetanib (60 μM) for 16 h. As shown in Figure 2B,C, a remarkable 10-fold reduction in intracellular RSV RNA levels and a concurrent 5-fold decrease in extracellular RSV RNA levels were evident upon vandetanib treatment. The plaque assay was performed to quantify infectious particles, comparing samples subjected to an EGFR inhibitor during RSV infection with samples containing solely viruses. This result revealed a notable approximately 10-fold reduction in the number of infectious viruses within the EGFR inhibitor-treated samples during RSV infection (Figure 2D). These findings strongly imply substantial attenuation of viral replication within host cells or a reduction in the production of infectious viral particles. These discernible reductions in both the intracellular and extracellular RSV RNA levels support the conclusion that vandetanib acts as a therapeutic agent against RSV infection. Furthermore, these results suggest that vandetanib inhibits viral replication within host cells and limits the subsequent release of infectious viral particles, highlighting its potential as a therapeutic agent against RSV infection.

### 2.3. RSV Activates EGFR Mediated Cell Survival Signal Cascade

**Figure 3:**
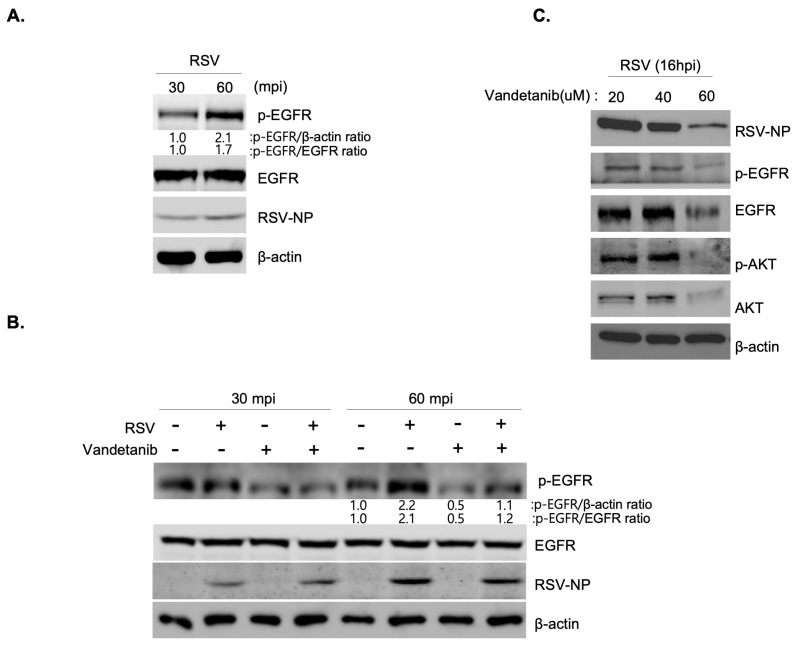
RSV activates an EGFR signal cascade. (**A**,**B**) Western blot showing an increase in p-EGFR protein expression in RSV (MOI 0.1)-infected cells at 30 and 60 min post-infection. (**A**) Rescue of RSV-induced activation of EGFR (p-EGFR) by vandetanib treatment (60 μM) (**B**). Whole cell lysates of RSV infected cells analyzed by Western blotting with antibodies specific to p-EGFR, EGFR, RSV-NP, and β-actin. β-actin was used as an internal loading control. (**C**) The decrease in EGF-mediated signal cascade in RSV (MOI 0.1)-infected cells with vandetanib treatment (60 μM) observed by Western blot analysis.

The EGFR signaling pathway is activated in response to stress-induced signals such as viral infections. EGFR signaling is activated by EGFR phosphorylation, which leads to a cascade of downstream signaling events, such as AKT phosphorylation [13,21,22]. To investigate whether EGFR-mediated cell survival signaling was triggered during RSV infection, we performed Western blot analysis. The results demonstrated a gradual 2.1-fold (p-EGFR/β-actin ratio) and 1.7-fold (p-EGFR/EGFR ratio) increase in the expression of phosphorylated EGFR (p-EGFR) protein in RSV-infected cells (MOI 0.1) at 30 and 60 min post-infection (Figure 3A). As shown in Figure 3B, although the overall expression of EGFR remained consistent before and after drug treatment, a reduction in p-EGFR expression was observed at 60 min post-infection. This result suggested that vandetanib treatment rescued the RSV-induced activation of EGFR, as evidenced by the decrease in p-EGFR expression. Further analysis of EGFR-mediated cascade proteins at varying concentrations of vandetanib (ranging from 20 to 60 μM) during MOI 0.1 RSV infection for 16 h post-infection revealed a significant decrease in the expression of RSV-NP with vandetanib treatment (60 μM) (Figure 3C). Additionally, treatment with 60 μM vandetanib inhibited the activation of downstream proteins, including p-EGFR and p-AKT. The stability of EGFR was decreased by inhibition of RSV replication when exposed to vandetanib for 16 h post-infection. Collectively, these results indicate that RSV activates an EGFR-mediated cell survival signaling cascade, and that the observed effects are rescued by vandetanib treatment.

### 2.4. RSV Promotes the Activation of EGFR and Generates RSV NP/EGFR/Tom20 Complex on the Mitochondria

**Figure 4:**
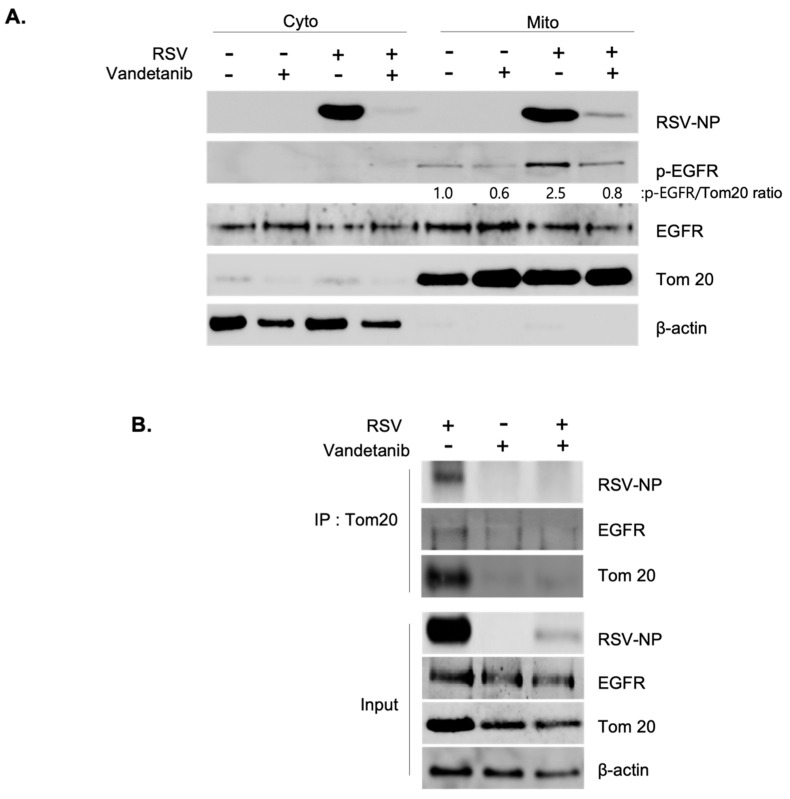

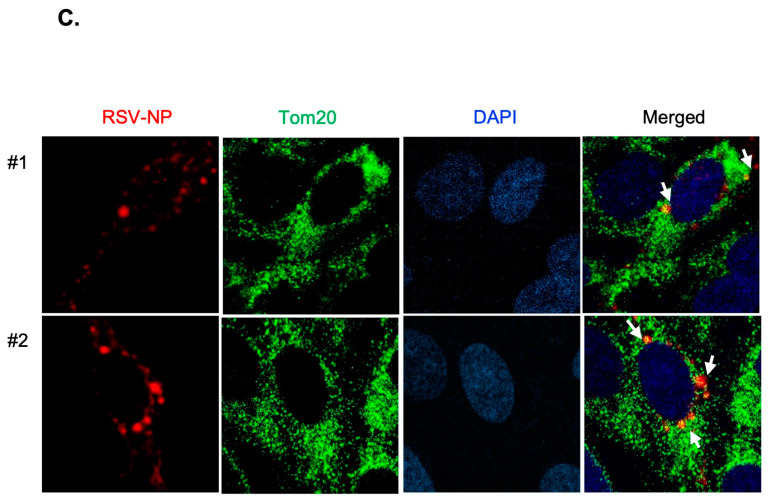
RSV activates EGFR and induces RSV-NP/EGFR/Tom20 complex on the mitochondria. (**A**) RSV-induced mitochondrial activation of EGFR. At 16 hrs post-infection, cytosolic and mitochondrial fractions isolated from uninfected and RSV-infected cells (MOI 0.1) in the presence of vandetanib (60 μM) or vehicle (DMSO) were evaluated by Western blotting with antibodies specific to p-EGFR, EGFR, and RSV-NP. Fractions: isolated cytosolic; Cyto: isolated mitochondria; Mito. Mitochondria markers: Tom20. (**B**) Immunoprecipitation (IP) determining the interaction between RSV-NP, EGFR, and Tom20. HEp-2 cells were infected with RSV (MOI 0.1) in the presence or absence of vandetanib (60 μM) for 16 hrs. IP was performed using anti-Tom20 antibody. The protein complexes were evaluated by Western blot analysis to detect the indicated proteins. (**C**) Confocal microscopy shows mitochondrial accumulation of RSV-NP in RSV-infected cells. RSV-infected HEp-2 cells were stained with RSV-NP (red) and Tom20 (green) antibodies. Nuclei, DAPI (blue); representative images of duplicate experiments, #1 and #2. Colocalization of Tom20 and RSV-NP antigen (yellow stain in merged image) is indicated by arrows (white) (630× magnification).

The presence of EGFR is not confined solely to the surface of the plasma membrane but also extends to intracellular organelle membranes and the nucleus. Specifically, EGFR is translocated to the mitochondria, where it regulates mitochondrial bioenergetics [4,15,23]. The interplay between EGFR activation and RSV infection was elucidated by isolating cytosolic and mitochondrial fractions from both uninfected and RSV-infected cells (MOI 0.1), treated with vandetanib (60 μM) or the vehicle (DMSO), followed by Western blot analysis. Our observations revealed the localization of RSV-NPs and EGFR to the mitochondria, with RSV-induced mitochondria-activated EGFR phosphorylation at 16 h post-infection. Notably, vandetanib treatment significantly reduced NP expression in both the cytosol and mitochondria, leading to a more than 3-fold decrease in viral p-EGFR within the mitochondria (Figure 4A). To investigate virus-induced mitochondrial complex formation, immunoprecipitation (IP) was performed using a translocase of the outer membrane 20 (Tom20) antibody (Figure 4B). Tom20 is located on the outer membrane and acts as an essential receptor for proteins targeted to the mitochondria. The mitochondrial protein import mechanism is not only a function of protein translocation but also deeply cooperates with the functional network of mitochondrial bioenergetics [24,25,26]. The results revealed that EGFR translocates to the mitochondria upon RSV infection, forming a complex with RSV-NPs and Tom20. Based on IP results, it is anticipated that the treatment with vandetanib has influenced the recognition ability of anti-Tom20 against Tom20, which is a mitochondrial membrane protein, making a complex with RSV-NP and EGFR. Consequently, our results have verified that an increase in the amount of pulled-down Tom20 correlates with a proportional increase in pulled-down NP. Consequently, it can be inferred that mitochondria proteins, Tom20, along with the viral protein NP and EGFR, will significantly contribute to the formation of the complex. This translocation and colocalization was further confirmed by an immunofluorescence assay (IFA). As shown in Figure 4C, the colocalization of RSV-NP (red) and Tom20 (green), in cells infected with RSV at an MOI of 0.1, was observed through confocal microscopy, evident as a yellow stain in the merged image.

## 3. Discussion

**Figure 5:**
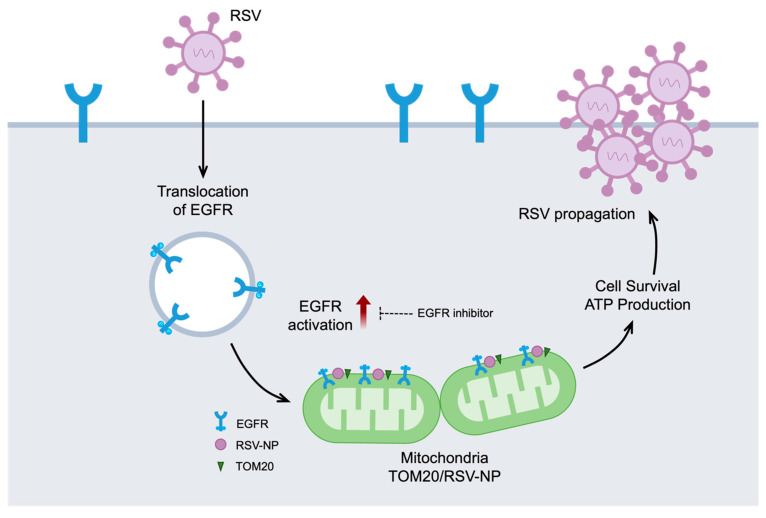
EGFR in mitochondria and RSV propagation. A schematic diagram showing the significant contribution of RSV-induced activation of EGFR and mitochondrial EGFR accumulation for viral propagation during the early stages of infection. RSV initiates the internalization of EGFR and subsequently promotes the translocation of activated EGFR into mitochondria. Treatment with vandetanib demonstrates a significant reduction in RSV replication and impedes the activation of EGFR. Within the mitochondria, a complex forms early in the virus infection process, involving the virus protein NP, EGFR, and the mitochondrial protein Tom20. This intricate interaction influences mitochondrial homeostasis, thereby contributing to ATP production and promoting cell survival, ultimately emphasizing its importance in virus propagation.

This study revealed that RSV activates an EGFR-mediated cell survival cascade during the early stages of infection and induces the translocation of EGFR to the mitochondria, where it maintains its activity. Vandetanib, an EGFR inhibitor, significantly reduces RSV replication, suggesting that EGFR is a promising target for RSV therapy. Furthermore, IP and confocal microscopy confirmed the formation of a complex between the viral protein NP, EGFR, and mitochondrial Tom20. This process, which is observed during the early stages after entry of viral infection, influences mitochondrial homeostasis and contributes to ATP production and cell survival. These processes underscore the critical role of EGFR-mediated signaling in viral propagation, emphasizing its importance during the early stages of RSV infection (Figure 5).

Recent studies have uncovered interesting connections between SARS-CoV-2 and the outer membrane protein of mitochondria, Tom70. The Orf9B protein of SARS-CoV-2 targets Tom70, which acts as a protein importer from the cytoplasm to the inner mitochondria and is important for the cellular antiviral response [27,28,29,30]. However, elucidation of EGFR’s functional role within mitochondria during SARS-CoV-2 infection remains a paramount subject for future investigation. This highlights the significance of unraveling the intricate interactions between mitochondria and respiratory viral infections, thus providing insights for enhanced comprehension of viral pathogenesis.

In the present study, we determined the antiviral efficacy of vandetanib against RSV infection. Another group investigated the role of EGFR in vaccinia virus (VACV) by utilizing EGFR inhibitors such as gefitinib, AG1478, and vandetanib. They defined the role of F1L, the mitochondria-targeted viral protein of VACV, as a Bcl-2 homolog that suppresses apoptosis. To induce cell survival and facilitate viral propagation during VACV infection, vaccinia growth factor (VGF), which shares a homology with epidermal growth factor, activates EGFR phosphorylation [31,32,33]. FDA-approved EGFR inhibitors, commonly employed as therapeutic agents for lung and thyroid cancers, can be successfully used to treat respiratory viruses. Discovering the intricacies of EGFR inhibitors against viruses can facilitate a straightforward approach for drug repurposing. Subsequent investigations must focus on elucidating the complex interplay between RSV, EGFR, and host immune responses, particularly emphasizing the role of mitochondria. This study aimed to unveil novel avenues for the development of targeted therapeutic strategies. We expect that further investigation of the role of EGFR in viral infections will not only enhance our understanding but also facilitate the development of innovative antiviral strategies.

## 4. Materials and Methods

### 4.1. Virus and Cell Culture

Respiratory syncytial virus (RSV) was provided by ATCC (American Type Culture Collection, Manassas, VA, USA) (VR-26). RSV was propagated and prepared in HEp-2 cells (ATCC). RSV infection was performed at a multiplicity of infection (MOI) of 0.1, under level 2 biosafety. Human epithelial Hep-2 cells were grown in DMEM/high-glucose (Hyclone, Logan, UT, USA) containing Pen strep and 10% fetal bovine serum (FBS) (Gibco, Grand Island, NY, USA). Cell lines were incubated in a humidified atmosphere at 37 °C and 5% CO_2_.

### 4.2. Reagents and Antibodies

The US Food and Drug Administration (FDA)-approved EGFR inhibitor vandetanib used in this study was purchased from Selleckchem. The primary antibodies used for the study were: rabbit polyclonal anti-EGFR (MERCK, Rahway, NJ, USA), rabbit monoclonal anti-phospho-EGFR (Y1068) (Abcam, Cambridge, UK), rabbit polyclonal anti-RSV nucleoprotein (Sino Biological, Beijing, China), rabbit monoclonal anti-Akt (Cell Signaling Technology, Danvers, MA, USA), rabbit monoclonal anti-phospho-Akt (Ser473) (Cell Signaling Technology), mouse monoclonal anti-β-actin (Cell Signaling Technology) and mouse monoclonal anti-Tom20 (BD). Secondary antibodies used for Western blot analysis were horseradish peroxidase-conjugated anti-mouse IgG (Invitrogen, Waltham, MA, USA) and horseradish peroxidase-conjugated anti-rabbit IgG (Invitrogen). Secondary antibodies used for immunofluorescence were Alexa Fluor 488 or 633 goat anti-mouse or anti-rabbit IgG (Life Technologies, Carlsbad, CA, USA).

### 4.3. Western Blot Analysis

For immunoblotting, whole-cell lysates were prepared by adding cell lysis buffer. Proteins were separated using SDS-PAGE, transferred onto PDF membranes, blocked with 5% skim milk in PBS containing 0.1% Tween-20 (PBS-T), and stained with antibodies against the indicated proteins. The membranes were washed five times with PBS-T and developed using Western ECL Femto Kit LPS Solution. ChemiDoc and ChemiDoc MP Imaging Systems (Bio-Rad, Hong Kong, China) were used for image capture and intensity control of protein expression.

### 4.4. Real-time qRT-PCR

Total RNA was extracted from the cells to analyze the expression levels of RSV genes using RNeasy Mini Kit (QIAGEN, Hong Kong, China) according to the manufacturer’s instructions. Extracellular viral RNA was isolated from the supernatants using the QIAamp Viral RNA Mini Kit (QIAGEN). The intracellular and extracellular RSV RNA copy numbers were determined by real-time qRT-PCR using a One-Step TB Green PrimeScript RT-PCR Kit Ⅱ (TaKaR, Hong Kong, China). The following primer sets were used for RT-PCR:

*RSV-NP* forward 5′-GGAACAAGTTGTTGAGGTTTATGAATATGC;
*RSV-NP* reverse 5′-TTCTGCTGTCAAGTCTAGTACACTGTAGT;
β-actin forward 5′-GATGACGAAGGAGATCACTG;
β-actin reverse 5′-CTGCTTGCTGATCCACAT.

### 4.5. Immunofluorescence

Cells grown on glass coverslips were infected with RSV at an MOI of 0.1. At 16 h post infection, the cells were fixed with 4% paraformaldehyde, blocked with a blocking buffer (2% BSA in PBS), and immunostained with the indicated antibodies. The coverslips were mounted in an Antifade Mounting Medium with DAPI (Vector Laboratories, Newark, CA, USA). Images were captured using a ZEISS LSM 900 confocal laser-scanning microscope (Carl Zeiss, Jena, Germany) and merged using the ImageJ software (v.1.53e).

### 4.6. Plaque Assay

The titer of infectious virus in the culture medium was determined by the plaque assay. The infectious cell culture medium was used to infect the HEp-2 cells, and the plates were incubated at 37 °C for 2 h. The virus incubation solution was replaced with medium containing 0.5% methylcellulose (Sigma, Kawasaki, Japan). After 72 h, the cells were fixed and stained with a 1% crystal violet solution in 50% ethanol (Sigma). The number of plaques was counted, and the titer was calculated in plaque-forming units per milliliter (PFU/mL).

### 4.7. Cell Viability Assay

Cell viability and ATP levels were measured by the CellTiter-Glo assay (Promega, Madison, WI, USA) using LUMIstar Omega (BMG LABTECH, Ortenberg, Germany) according to the manufacturer’s instructions. The total cell number and viability were quantified using a hemocytometer and the Trypan Blue dye exclusion method, respectively.

### 4.8. Subcellular Fractionation

Mitochondria were isolated using a mitochondrial isolation kit (Thermo Scientific, Waltham, MA, USA) according to the manufacturer’s instructions. Proteins extracted from each cellular fraction were analyzed by Western blotting using the indicated antibodies.

### 4.9. Statistical Analysis

Unpaired Student’s *t*-tests were performed using the GraphPad Prism 9 software (GraphPad Software, La Jolla, CA, USA).

## Data Availability

Data is contained within the article.
